# Response to pembrolizumab in a patient with xeroderma pigmentosum, metastatic cutaneous melanoma and multiple squamous cell carcinomas: a case report

**DOI:** 10.1093/skinhd/vzaf038

**Published:** 2025-09-02

**Authors:** Júlia C Martins, Abna F S Vieira, Olavo Feher

**Affiliations:** Instituto do Câncer do Estado de São Paulo, Universidade de São Paulo, São Paulo, Brazil; Instituto do Câncer do Estado de São Paulo, Universidade de São Paulo, São Paulo, Brazil; Instituto do Câncer do Estado de São Paulo, Universidade de São Paulo, São Paulo, Brazil

## Abstract

Xeroderma pigmentosum (XP) is a rare genetic disorder associated with defective DNA repair, leading to extreme ultraviolet sensitivity and a significantly elevated risk of malignancies. This case report details a patient with XP and metastatic melanoma who achieved complete clinical remission with pembrolizumab, highlighting the potential efficacy of immune checkpoint inhibitors in tumours with high mutational burdens. These findings underscore a promising therapeutic avenue for this vulnerable population with limited treatment options.

## Teaser

Xeroderma pigmentosum (XP) is a rare genetic disorder associated with defective DNA repair, leading to extreme UV sensitivity and a significantly elevated risk of malignancies. This case report details a patient with XP and metastatic melanoma who achieved complete clinical remission with pembrolizumab, highlighting the potential efficacy of immune checkpoint inhibitors in tumours with high mutational burdens. These findings underscore a promising therapeutic avenue for this vulnerable population with limited treatment options.

**What is already known about this topic?**

- Xeroderma pigmentosum (XP) is associated with defective DNA repair leading to extreme ultraviolet sensitivity and a very high risk of cutaneous neoplasias.

**What does this study add?**

- We report a patient with XP with numerous cutaneous and ocular malignancies, including metastatic melanoma diagnosed in 2014.
- Treatment with the immune checkpoint inhibitor (ICI) pembrolizumab for 1 year led to a rapid and complete response of the metastatic melanoma and also to a very dramatic regression of all cutaneous malignancies.
- In addition, a corneal conjunctival malignancy also regressed.
- No marked toxicity or immune-related diseases ensued with the ICI.
- Response was maintained 6 months post-ICI discontinuation.
- ICI anti-programmed cell death protein 1 shows marked activity in patients with XP and should be studied prospectively, perhaps earlier in these patients.

Xeroderma pigmentosum (XP) is a rare autosomal recessive genetic disorder characterized by defective DNA repair following ultraviolet (UV) damage. This defect leads to extreme sensitivity to UV radiation and a dramatically increased risk of developing cutaneous malignancies.^1^ Individuals with XP face a 2000-fold increased risk of melanoma and a staggering 10 000-fold increased risk of basal and squamous cell carcinomas compared with the general population.^2^ Advanced skin cancers are a leading cause of mortality in this population.^2^ While carcinomas typically arise during childhood or adolescence in UV-exposed areas and often present as multiple lesions, melanomas occur in 3–50% of cases,^2,3^ exhibit a higher metastatic potential and are associated with worse prognosis.^1^ Treatment options for advanced or metastatic XP-associated malignancies are limited,^4^ and cytotoxic chemotherapy often causes significant toxicity in this population.^5^

XP-associated skin cancers have a high mutational burden,^3^ a predictive factor for response to immune checkpoint inhibitors (ICIs).^6^ ICIs have demonstrated remarkable antitumour efficacy in various malignancies, including advanced cutaneous melanoma and squamous cell carcinomas.^7^ Nonetheless, evidence regarding the use of ICIs in XP-associated advanced cancers, particularly metastatic melanoma, remains limited.^4,8–10^

## Case report

We report the case of a dramatic response to pembrolizumab in a patient with XP, metastatic cutaneous melanoma and numerous skin carcinomas.

The patient, a woman diagnosed with XP in childhood, has been under care at the Hospital das Clínicas, University of São Paulo (HCFMUSP), since 1998. Her medical history includes multiple cutaneous and ocular malignancies.

In 2013, the patient presented with a pigmented lesion on her scalp, which was surgically excised in June. Histopathological analysis revealed nodular malignant melanoma with a Breslow thickness of 14 mm, subcutaneous invasion, ulceration and positive margins, consistent with stage pT4b disease. In August of the same year, she underwent margin expansion surgery, revealing no viable neoplasia. Concurrently, partial glossectomy was performed to treat a squamous cell carcinoma of the tongue.

In 2014, metastatic melanoma was confirmed in a left axillary lymph node. A positron emission tomography and computed tomography (PET-CT) scan revealed multiple subcutaneous nodules suggestive of disease implants. Given the unresectable lesions, systemic chemotherapy with cyclophosphamide, vincristine and dacarbazine (CVD regimen) was initiated. Partial treatment response was achieved, but treatment was discontinued due to severe toxicities, including febrile neutropenia, gastrointestinal symptoms and septic shock.

**Figure 1:**
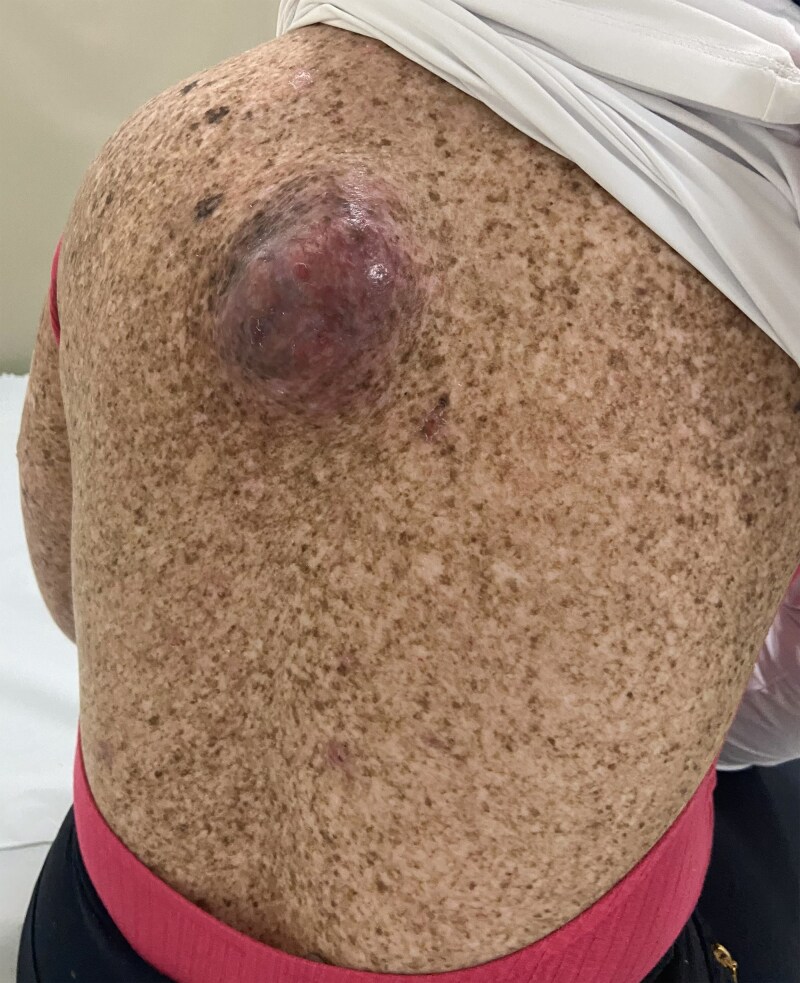
Dorsal nodule consistent with metastatic cutaneous melanoma and xeroderma pigmentosum-related hyperpigmented skin lesions before treatment with pembrolizumab.

Between 2018 and 2022, the patient experienced progressive left axillary lymphadenopathy and the emergence of nodules in the thoracic wall and right breast. Axillary lymphadenectomy confirmed melanoma metastases. She remained under regular follow-up until 2023, when a large dorsal nodule emerged (Figure 1). Biopsy confirmed metastatic melanoma, and PET-CT revealed multiple subcutaneous nodules and a liver nodule in segment VI, suggestive of metastasis. Additionally, the patient exhibited multiple cutaneous lesions suggestive of carcinomas, primarily on the trunk and back, with some also affecting the face, scalp and limbs. While not all these lesions were biopsied, prior histopathological analyses had confirmed malignancy in some of them. Multidisciplinary evaluation deemed the lesions unresectable, and systemic treatment was recommended.

**Figure 2:**
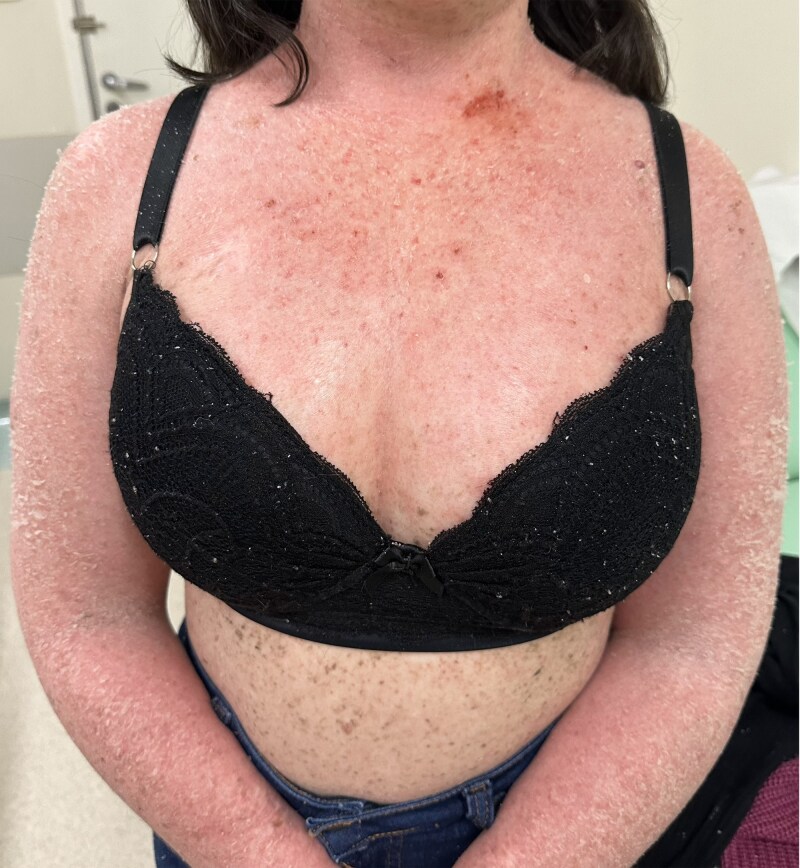
Rash and skin peeling during treatment with pembrolizumab.

**Figure 3:**
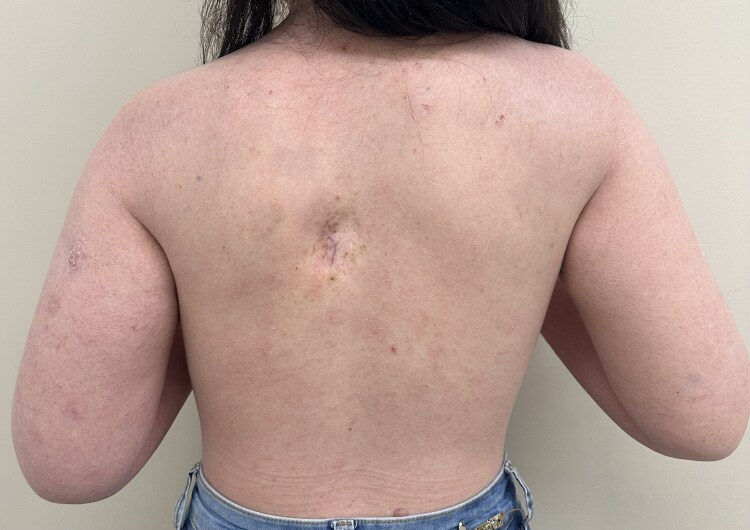
Resolution of dorsal nodule and hyperpigmented skin lesions after 17 cycles of pembrolizumab.

The patient was included in an institutional clinical trial evaluating the monoclonal antibody anti-programmed cell death protein 1 (anti-PD1) pembrolizumab for a maximal duration of 1 year, or 17 cycles (as opposed to the standard 2 years). It is important to note that immunotherapy is not yet available routinely for any indication in the Brazilian public health system. Following pembrolizumab initiation, the patient exhibited excellent tolerance, with mild toxicity limited to rash and skin peeling, managed conservatively. A notably rapid regression of the metastatic melanoma lesion was observed after only two doses of pembrolizumab. This early response was subsequently followed by a progressive remission of the numerous cutaneous lesions suggestive of carcinomas, as well as XP-associated hyperpigmented skin lesions. After 17 cycles, the patient achieved complete clinical remission of the dorsal metastatic melanoma (Figures 2, 3). Additionally, radiological resolution of subcutaneous nodules and hepatic lesion was observed. Recent imaging, conducted 6 months after the suspension of pembrolizumab, confirmed sustained complete response, and the patient will continue undergoing follow-up assessments per the clinical trial protocol.

## Discussion

A literature review identified two previously reported cases on the use of immunotherapy in patients with metastatic melanoma and XP.^8,9^ One case described a complete clinical response, with significant regression of pulmonary metastases after 3 months of anti-PD1 therapy,^9^ while the other reported a partial response of melanoma metastases and regression of cutaneous carcinomas after four cycles of pembrolizumab.^8^ Additionally, a recent retrospective review of patients with XP treated with ICIs identified four cases of metastatic melanoma that had not been previously reported in the literature.^10^ Among these, two patients achieved complete responses – one after 4 months of anti-PD1 treatment and another without a specified time to response – while the remaining two showed objective responses, including one with clinical response after three cycles and radiological response after six cycles of immunotherapy. These findings align with our case, in which the patient demonstrated regression of the melanoma lesion after two cycles of pembrolizumab, followed by gradual resolution of multiple skin carcinomas, ultimately achieving complete remission after 17 treatment cycles.

Despite the potential risks associated with ICIs, including severe autoimmune complications, our patient did not experience significant immune-related toxicity. Although DNA repair deficiency in patients with XP raises concerns about potential long-term immune toxicity, no increased risk has been reported in the few available cases. Nevertheless, long-term safety data for ICIs in patients with XP are still lacking.

This case highlights the potential efficacy of ICIs in treating advanced malignancies in XP, where the high mutational burden may enhance sensitivity to immunotherapy. Pembrolizumab led to a rapid and complete clinical and radiological remission without significant adverse effects, reinforcing its role as a promising therapeutic option for this vulnerable population. Although combination regimens such as anti-PD1 with anti-cytotoxic T-lymphocyte antigen 4 typically show higher response rates,^7^ their benefit in XP remains unclear – particularly given the apparent heightened sensitivity to monotherapy seen in our case and in previous reports.^8–10^ In this context, the incremental efficacy of combined therapy is uncertain, whereas the increased risk of toxicity and higher cost is well established. This case contributes to a growing body of evidence supporting the use of ICIs in tumours with high mutational burdens, offering hope for improved outcomes in patients with limited therapeutic options. While further research is needed to fully assess the role of immunotherapy in metastatic melanoma associated with XP, the rarity of the disease and the promising responses observed suggest that immunotherapy should be considered a standard treatment approach in these patients.

## Data Availability

The data underlying this article will be shared on reasonable request to the corresponding author.
